# Exploring the Nutraceutical Potential of Extra Virgin Olive Oils From Sicilian Well‐Known and Lesser‐Known Cultivars

**DOI:** 10.1002/cbdv.202503286

**Published:** 2026-04-27

**Authors:** Ali Mert Yetisgin, Giovanni Bartolomeo, Nicola Cicero, Nicola Micale, Rosaria Costa, Mariateresa Cristani

**Affiliations:** ^1^ Faculty of Pharmacy İnönü University Battalgazi Malatya Türkiye; ^2^ Department of Biomedical Dental, Morphological and Functional Images Sciences (BIOMORF) University of Messina Messina Italy; ^3^ Science4Life s.r.l. Start‐Up of the University of Messina Messina Italy; ^4^ Department of Chemical Biological, Pharmaceutical and Environmental Sciences University of Messina Messina Italy

**Keywords:** anti‐inflammatory, antioxidant, cultivars, extra virgin olive oil, polyphenols

## Abstract

This study examined the health benefits of extra virgin olive oils (EVOOs) from three different Sicilian cultivars, namely Nocellara del Belice, Ogliarola Messinese, and the lesser‐known Verdello, grown in the regions of Agrigento and Messina. The quality parameters of the EVOOs, including free acidity, peroxide value, oxidation indices, and fatty acid composition, were first evaluated. Subsequently, the hydroalcoholic extracts of these oils (EVOOEs) were examined and their content of polyphenols was determined using HPLC and UV–vis spectrophotometry, as well as their antioxidant capacity (DPPH, ABTS, ORAC, and FRAP tests) and anti‐inflammatory properties (COX‐1/COX‐2 inhibition assay) were also determined. The results showed significant qualitative and quantitative differences between cultivars: Nocellara and Verdello showed, in order, a higher content of both polyphenols (phenolic acids + flavonoids) than Ogliarola, correlated with a higher antioxidant activity profile and greater health‐promoting potential. In addition, all extracts showed modest inhibition of COX‐1 compared to COX‐2, indicating potential selective anti‐inflammatory effects. These results highlight the nutritional value of Sicilian EVOOs and the importance of cultivar selection. They also underscore the contribution of Sicilian olive oil production to dietary and therapeutic applications.

## Graphical abstract

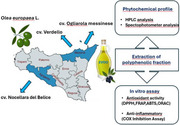

**Abbreviations**
5‐LOX: 5‐Lipoxygenase
ANOVA: One‐way Analysis Of Variance
COU: *p*‐Coumaric acid
COX: Cyclooxygenase
EVOOEs: Extra virgin olive oils extracts
EVOOs: Extra virgin olive oils
NF‐κB: Kappa‐light‐chain‐enhancer of activated B cells
Nrf2: Nuclear factor erythroid 2‐related factor 2
NSAIDs: Nonsteroidal Anti‐Inflammatory Drugs
OAC: Oleacein
OCA: Oleocanthal
OEP: Oleuropein
OHT: Hydroxytyrosol
PLA2: Phospholipase A2
ROS: Reactive oxygen species
SYR: Syringic acid
TYR: Tyrosol
VAN: Vanillic acid

## 1 Introduction

In the intricate biochemical landscape of the human body, there is a delicate balance between oxidants and antioxidants. Endogenous oxidants, particularly reactive oxygen species (ROS), are by‐products of cellular metabolism and immune response [1]. They include radical (·O_2_^–^, ·OH) and non‐radical species (H_2_O_2_, ^1^O_2_), both highly reactive, that can attack essential biomacromolecules such as DNA, lipids, and protein causing mutations or impairing functions. While essential for various physiological functions, an imbalance of the ration oxidants/antioxidants favoring ROS can lead to oxidative stress, a condition implicated in the pathogenesis of numerous chronic diseases, including cardiovascular disorders, neurodegenerative conditions, and certain types of cancer [2]. To counteract oxidative damage, the body relies on an array of endogenous antioxidants [3]. However, dietary intake of natural antioxidants, especially those abundant in plant‐based foods, can significantly boost this innate defense system. Among these, polyphenols, bioactive compounds found in various plant sources, have earned attention for their potent antioxidant and anti‐inflammatory properties [4]. Flavonoids, a subclass of polyphenols, are particularly notable for their widespread presence in the human diet and their substantial contribution to antioxidant intake.

The Mediterranean diet, characterized by its high content of fruits, vegetables, fish, legumes, whole grains, and olive oil, is renowned for its health‐promoting effects. Olive oil, especially EVOO, serves as a primary fat source in this dietary pattern. In addition to monounsaturated fatty acids, EVOO is rich in polyphenols such as hydroxytyrosol (OHT), tyrosol (TYR), and oleuropein (OEP), which are credited with various health benefits [5]. Extensive research underscores the multifaceted health benefits of EVOO, particularly due to its antioxidant and anti‐inflammatory effects. These properties are largely attributed to its polyphenolic content, which not only scavenges free radicals but also modulates key cellular pathways involved in oxidative stress and inflammation. For example, polyphenols found in EVOO have been shown to activate nuclear factor erythroid 2‐related factor 2 (Nrf2), a transcription factor that promotes the expression of antioxidant enzymes while simultaneously suppressing nuclear factor kappa B (NF‐κB), a key regulator of inflammation [6]. Additionally, specific bioactive compounds in EVOO contribute to its anti‐inflammatory effects. Oleacein (OAC), for instance, can inhibit critical inflammatory enzymes such as phospholipase A2 (PLA2) and 5‐lipoxygenase (5‐LOX). Another compound, oleocanthal (OCA), exhibits anti‐inflammatory activity comparable to that of nonsteroidal anti‐inflammatory drugs (NSAIDs) like ibuprofen by inhibiting cyclooxygenase enzymes (COX‐1 and COX‐2), thereby reducing the production of pro‐inflammatory prostaglandins. This dual action of combating oxidative stress and inflammation places EVOO as a potent dietary agent in the prevention and management of various chronic diseases [7_,_ 8]. Therefore, EVOO stands out not only as a culinary product, but also as a functional food with significant health‐promoting properties.

The Oleaceae family includes approximately 30 genera and 600 species adapted to different climates worldwide. Within the species *Olea europaea* L., numerous cultivars have been developed, particularly in southern Italy, such as in Sicily, where unique pedoclimatic traits have a strong influence on the quality of olive oil. The excellent nutritional properties of EVOO obtained from this species are liked to its high content of monounsaturated fats and polyphenols, which reduce risk of developing atherosclerosis, improve cerebral circulation, and protect against from oxidative stress in regular consumers. In Sicily, the cultivation of olive varieties such as Nocellara del Belice, Biancolilla, and Cerasuola, has led to the production of EVOOs with distinct organoleptic profiles and varying polyphenolic contents [9, 10, 11]. Most studies on Sicilian EVOOs reported in the literature emphasize the value of their polyphenolic fractions and antioxidant properties. In this regard, the primary objectives of this study were to investigate not only the pool of antioxidants, but also the anti‐cyclooxygenase effects of EVOOs produced in Sicily from the well‐known cultivars Nocellara del Belice and Ogliarola Messinese, and from the lesser‐known cultivar Verdello. This last variety appears to have been little studied, as certain critical issues, including climate change impact (prolonged droughts and increased temperatures), soil degradation, changes in agricultural practices (e.g., mechanization and increased use of fertilizers and pesticides), socioeconomic factors and introduction of new pests/diseases, have almost halted its cultivation. By analyzing their composition and testing their bioactivity, the present research aimed to elucidate the potential health benefits associated with the consumption of these EVOOs. In addition, due to the presence of Verdello among the investigated cultivars, the study was meant to highlight the significance of preserving and promoting the unique olive biodiversity of Sicily, emphasizing the health‐promoting anti‐inflammatory, antioxidant, cultivars, extra virgin olive oil, polyphenols properties of its EVOOs and their role in the Mediterranean diet.

## 2 Results

### 2.1 Quality Parameters of EVOOs

#### 2.1.1 Physicochemical Quality

The free acidity value was calculated and expressed as oleic acid content per 100 g of oil (Table 1). According to EU Regulation 2022, Nocellara and Verdello can be classified as EVOOs, whereas Ogliarola slightly exceeds the maximum limit set for EVOO, which is <0.8 [12]. Besides free acidity, other parameters studied were peroxide value, and the oxidation level indices K232, K270, and Δ*K*, all reported in Table 1 as well. From the values reported and included in the prescribed ranges, all the three oils can be safely categorized as extra virgin.

**TABLE 1:** Free acidity, peroxide value, and oxidation indices of olive oil samples.

Cultivar	Free acidity (%)	Peroxide value (PV) (meq O_2_ kg^−1^)	K232	K270	Δ*K*
Nocellara del Belice	0.51 (0.8)	6.7 (≤20)	1.67 (≤2.5)	0.11 (≤0.22)	0.001 (≤0.01)
Verdello	0.79 (0.8)	10.6 (≤20)	1.91 (≤2.5)	0.15 (≤0.22)	0.002 (≤0.01)
Ogliarola Messinese	0.82 (0.8)	8.4 (≤20)	1.75 (≤2.5)	0.14 (≤0.22)	0.001 (≤0.01)

*Note*: EU Regulation ranges for EVOO are shown in brackets [12].

### 2.2 Fatty Acid Composition of EVOO Samples

**FIGURE 1:**
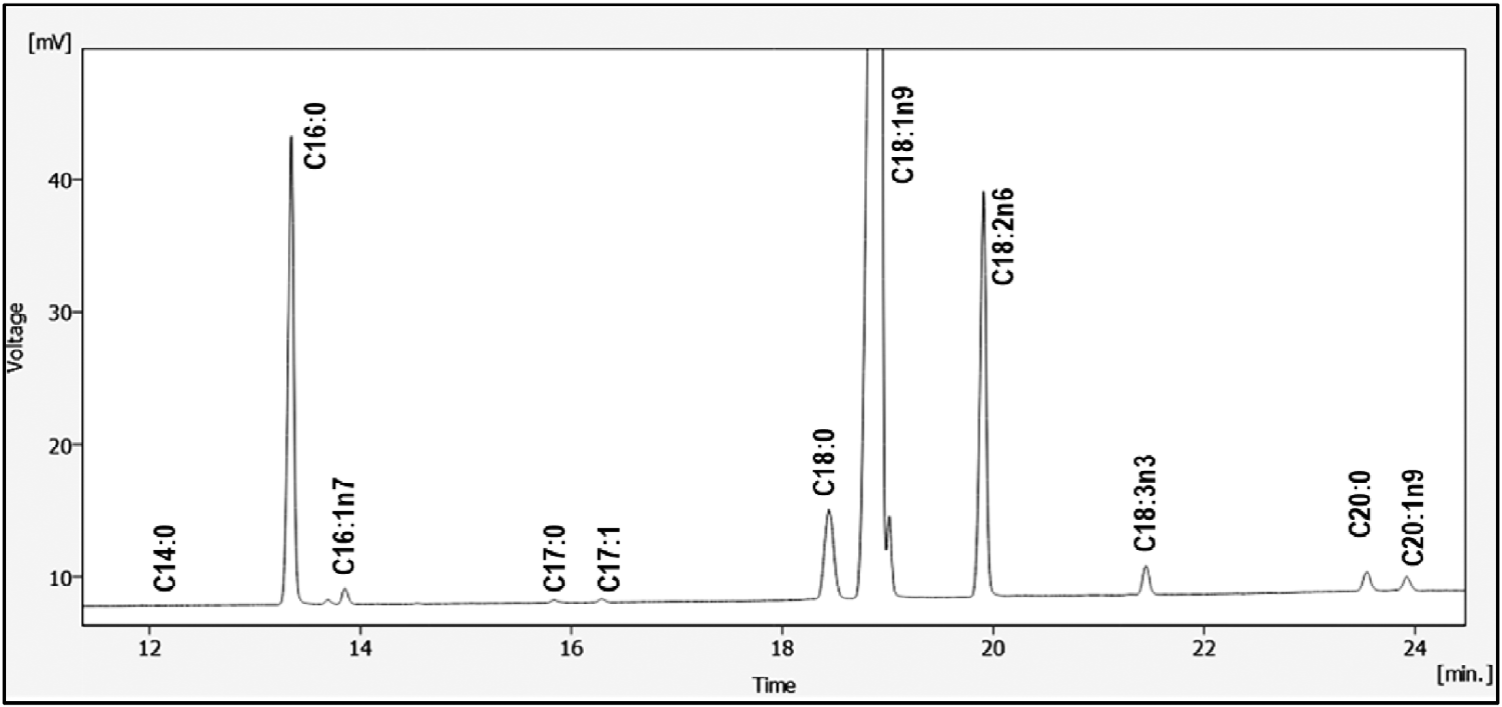
GC‐FID chromatogram of the fatty acid in an EVO sample (Ogliarola Messinese, *n* = 3).

The quali‐quantitative composition of fatty acids has been reported in Table 2 and illustrated in Figure 1, where a uniform distribution of analytes in the three cultivars can be observed. Predominant fatty acids were oleic (C18:1n9), followed by palmitic (C16:0) and linoleic (C18:2n6) acids. Because of this, mono‐unsaturated fatty acids represented the most abundant acidic fraction. Each fatty acid content (%) fell within the ranges prescribed by EU Regulation for EVOOs.

**TABLE 2:** Fatty acid composition of the EVOO samples.

Fatty acid	Nocellara del Belice	Ogliarola Messinese	Verdello	EVOO range [12]
C14:0	0.01 ± 0.00	0.01 ± 0.00	0.01 ± 0.00	≤0.03
**C16:0**	**10.79 ± 0.88**	**13.91 ± 0.91**	**11.87 ± 0.73**	**7–20**
C16:1n7	0.44 ± 0.09	1.08 ± 0.32	1.18 ± 0.21	0.3–3.5
C17:0	0.05 ± 0.00	0.16 ± 0.02	0.18 ± 0.03	≤0.4
C17:1n7	0.07 ± 0.01	0.25 ± 0.03	0.22 ± 0.05	≤0.6
C18:0	3.02 ± 0.21	3.52 ± 0.68	1.92 ± 0.12	0.5–5.0
**C18:1n9**	**74.01 ± 2.01**	**69.72 ± 1.88**	**72.75 ± 2.15**	**55–85**
**C18:2n6**	**9.87 ± 0.98**	**9.48 ± 0.57**	**10.81 ± 0.68**	**2.5–21.0**
C18:3n3	0.73 ± 0.05	0.91 ± 0.07	0.49 ± 0.06	≤1.00
C20:0	0.53 ± 0.06	0.55 ± 0.02	0.32 ± 0.02	≤0.6
C20:1n9	0.45 ± 0.07	0.38 ±0.03	0.22 ± 0.01	≤0.5
C22:0	0.02 ± 0.00	0.02 ± 0.00	0.02 ± 0.00	≤0.2
C24:0	0.01 ± 0.00	0.01 ± 0.00	0.01 ± 0.00	≤0.2
*Σ SFA*	*14.43 ± 1.15*	*18.18 ± 1.63*	*14.33 ± 0.90*	
*Σ MUFA*	*74.97 ± 2.18*	*71.43 ± 2.26*	*74.37 ± 2.42*	
*Σ PUFA*	*10.60 ± 1.03*	*10.39 ± 0.64*	*11.30 ± 0.74*	

*Note*: Values are expressed as GC peak area % and are means of triplicate injections.

### 2.3 Polyphenolic Composition of EVOOEs

#### 2.3.1 Total Phenolic and Flavonoid Content of EVOOEs

The results of the experiments relating to the total content of phenols and flavonoids are summarized in Table 3. In general, in terms of phenolic content, the results range from 85.4 µg GAE mg^−1^ to 135.3 µg GAE mg^−1^, with Nocellara and Verdello showing a similar and higher phenolic content than Ogliarola. As for flavonoid content, the values range from 380.8 µg rutin mg^−1^ to 988.3 µg rutin mg^−1^. Again, Nocellara and Verdello have higher levels than Ogliarola, although they are less comparable to each other.

**TABLE 3:** Total phenolic and flavonoid content of EVOOEs as measured by means of spectrophotometric methods.

EVOOEs	Folin–Ciocalteu µg GAE mg^−1^ ±SD^*^	Flavonoids µg rutin mg^−1^ ±SD
Nocellara	135.3 ± 10.8^a^	873.1 ± 34.3^a^
Ogliarola	85.4 ± 4.6^b^	380.8 ± 38.6^b^
Verdello	130.6 ± 5.7^a^	988.3 ± 10.3^c^

*Note*: For each column, means with the same letter are not significantly different for each other (*p* > 0.05).

* GAE: Gallic acid equivalent.

#### 2.3.2 HPLC Analysis

**FIGURE 2:**
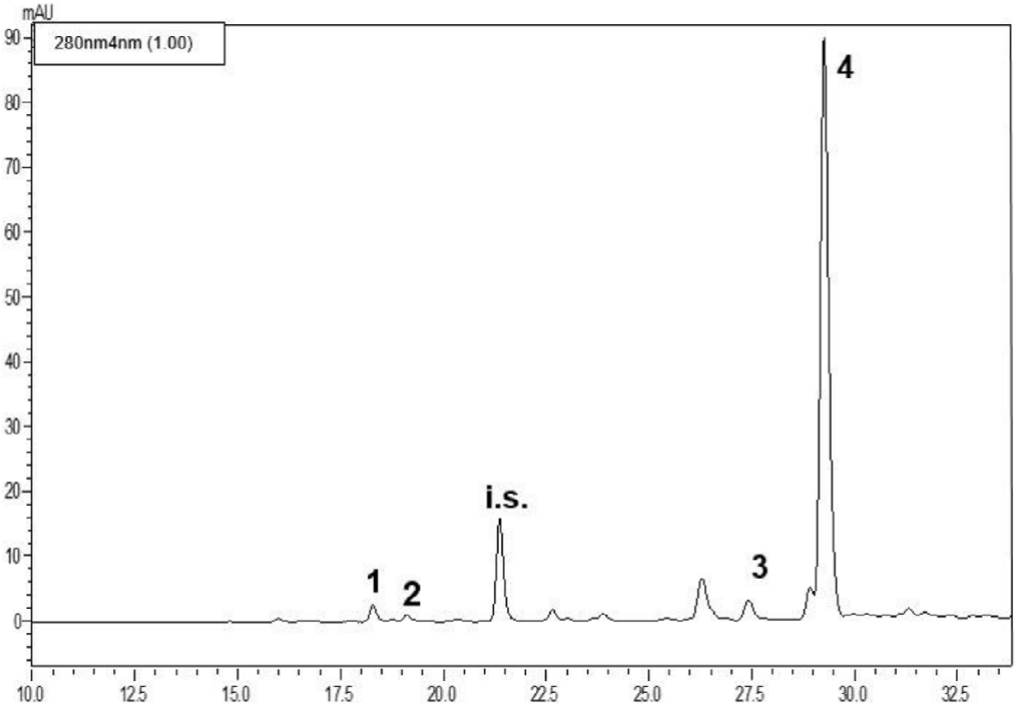
LC‐PDA chromatogram of the methanolic extract obtained from the cultivar Ogliarola Messinese (*n* = 3). See Table 4 for peak assignment; i.s.: syringic acid (internal standard).

**FIGURE 3:**
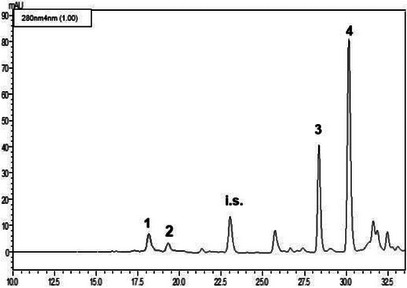
LC‐PDA chromatogram of the methanolic extract obtained from the cultivar Nocellara del Belice (*n* = 3). See Table 4 for peak assignment; i.s.: syringic acid (internal standard).

**FIGURE 4:**
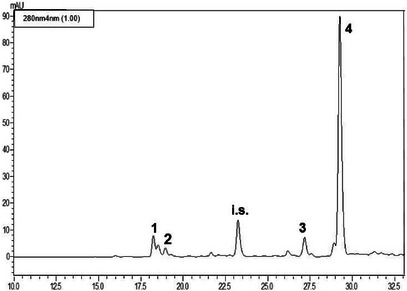
LC‐PDA chromatogram of the methanolic extract obtained from the cultivar Verdello (*n* = 3). See Table 4 for peak assignment; i.s.: syringic acid (internal standard).

The LC‐PDA analyses (see Figures 2, 3, 4) of the methanolic extracts revealed the presence of some phenolic compounds, namely the phenylethanoids hydroxytyrosol and tyrosol and the secoiridoids oleacein and oleocanthal. Data reported in Table 4 reveal notable differences in the phenolic profiles: Nocellara del Belice exhibited the highest concentration of OHT (5.23 mgL^−1^), followed by Verdello (3.82 mgL**^−^** ^1^); Ogliarola reported the lowest amount of OHT (1.93 mgL^−1^). Verdello had the highest TYR level (4.28 mgL^−1^), slightly above Nocellara (3.17 mgL^−1^). Ogliarola again showed the lowest content of phenolic compound (2.52 mgL^−1^), with greater variability as indicated by the standard deviation. As regards OAC, Nocellara del Belice showed a markedly higher concentration (65.20 mgL^−1^), more than double that of Verdello (27.33 mgL^−1^) and Ogliarola (25.43 mgL^−1^). Finally, OCA was the predominant polyphenol in all three cultivars, with Nocellara having the maximum amount (195.56 mgL^−1^), followed by Verdello (183.21 mgL^−1^) and Ogliarola (177.31 mgL^−1^).

**TABLE 4:** Phenolic composition of the methanolic extracts obtained from the three cultivars of extra virgin olive oil.

EVOOEs	OHT (µg mL^−1^) ± SD	TYR (µg mL^−1^) ± SD	OAC (µg mL^−1^) ± SD	OCA (µg mL^−1^) ± SD
Nocellara	5.23 ± 0.32 ^a^	3.17 ± 0.24 ^a^	65.20 ± 1.02 ^a^	195.56 ± 1.29 ^a^
Ogliarola	1.93 ± 0.19 ^b^	2.52 ± 0.62 ^b^	25.43 ± 0.41 ^b^	177.31 ± 2.04 ^b^
Verdello	3.82 ± 0.26 ^c^	4.28 ± 0.15 ^c^	27.33 ± 0.85 ^b^	183.21 ± 1.99 ^b^

Abbreviations: OAC, oleacein; OCA, oleocanthal; OHT, hydroxytyrosol; TYR, tyrosol.

The results represent the mean ± SD of three independent experiments in triplicate (*n* = 3). a–c: Different superscript letters in the same column indicate significantly different values for a given parameter (*p* < 0.05 by post hoc Tukey's HSD test).

### 2.4 Antioxidant Capacity

Whole, the results from all four tests consistently highlight the significant antioxidant capacity of olive oil extracts, primarily attributed to their polyphenolic composition. The use of multiple assays provided a more comprehensive understanding of their antioxidant behavior across different oxidative environments.

The results from the DPPH and FRAP assays ranged from 0.11 µmol TE mg^−1^ to 0.43 µmol TE mg^−1^ and 0.78 µmol Fe^2+^E mg^−1^ to 1.61 µmol Fe^2+^E mg^−1^, respectively; Verdello exhibited values close to Nocellara in both tests, with a slight predominance over it and high antioxidant activity compared to Ogliarola (Table 5).

**TABLE 5:** Antioxidant activity the olive oil phenolic fraction extracts measured by means of four in vitro tests.

EVOOEs	DPPH µmol TE mg^−1^ ± SD	FRAP µmol Fe^2+^E mg^−1^ ± SD	ABTS µmol TE mg^−1^ ± SD	ORAC µmol TE mg^−1^ ± SD
Nocellara	0.40 ± 0.04^a^	1.49 ± 0.10^a^	0.67 ± 0.16^a^	25.63 ± 6.96^a^
Ogliarola	0.11 ± 0.00^b^	0.78 ± 0.06^b^	0.44 ± 0.14^b^	6.07 ± 1.05^b^
Verdello	0.43 ± 0.03^a^	1.61 ± 0.09^a^	0.63 ± 0.10^a^	14.88 ± 4.09^c^

*Note*: The results represent the mean ± SD of three independent experiments in triplicate (*n* = 3). For each essay, means with the same letter are not significantly different for each other (*p >* 0.05).

Abbreviations: Fe^2+^E, Ferrous equivalents; TE, Trolox equivalents.

As regards the ABTS test, the results ranged between 0.44 µmol TE mg^−1^ to 0.67 µmol TE mg^−1^, with Nocellara showing slightly higher values than Verdello, and demonstrating higher antioxidant activity than the Ogliarola samples. Finally, in the ORAC test, the results ranged from 6.07 µmol TE mg^−1^ to 25.63 µmol TE mg^−1^; the Nocellara variety showed significantly higher efficacy than Verdello, while Ogliarola exhibited lower efficacy than both Verdello and Nocellara [13].

A correlation among the different parameters was investigated in particular, to analyze how the differences recorded by antioxidant activity values determined by different tests can be explained on the basis of the concentrations of the individual phenolic compounds. Results showed a very good positive and significant correlation between concentration of OHT and TYR antioxidant activity determined by the four assays (Table 6). The high antioxidant activity of these compounds has been demonstrated and described in the literature [14]. A good positive and significant correlation even if with lower values was determined also between the concentration OCA and OAC (Table 6).

**TABLE 6:** Pearson correlation coefficients of phenolic compounds concentration and antioxidant activity values.

	DPPH	FRAP	ABTS	ORAC
Flavonoids	1^a^	1^a^	1^a^	0.72^a^
Hydroxytyrosol	0.87^a^	0.84^a^	0.80^a^	0.99^a^
Tyrosol	0.83^a^	0.86^a^	0.89^a^	0.31
Oleacein	0.43	0.42	0.36	0.91^a^
Oleocanthal	0.68^b^	0.65^b^	0.60^b^	0.99^a^

^a^ Significant correlation with *p* < 0.05.

^b^ Significant correlation with *p* < 0.01.

### 2.5 COX‐1 and COX‐2 Inhibitory Activity

The results of COX‐1 and COX‐2 inhibition by EVOOEs are shown in Table 7. Based on the data, the COX‐1 inhibition potency appears relatively consistent across the different cultivars. In contrast, COX‐2 inhibition shows a progressive increase across the cultivars, following the order: Ogliarola < Nocellara < Verdello. This trend is further supported by the selectivity index (SI), calculated as the ratio of IC_50_ for COX‐1 to IC_50_ for COX‐2. Both Nocellara and Verdello cultivars demonstrated a higher selectivity for COX‐2 over COX‐1, indicating a preferential inhibitory effect on the COX‐2 isoform (Table 7).

**TABLE 7:** Percentage of inhibition and IC_50_ values of extracts against COX‐1 and COX‐2.

EVOOEs	% inh. COX‐1	% inh. COX‐2	IC_50_ COX‐1 µg mL^−1^	IC_50_ COX‐2 µg mL^−1^	SI
Nocellara	21.60 ± 0.07^a^	57.08 ± 0.5^a^	92.64 ± 0.67^a^	35.04 ± 0.15^a^	2.6
Ogliarola	24.00 ± 0.04^b^	43.70 ± 0.9^b^	83.56 ± 0.44^b^	45.77 ± 0.08^b^	1.8
Verdello	25.30 ± 0.06^c^	61.20 ± 0.4^c^	78.53 ± 0.42^c^	32.68 ± 0.19^c^	2.4
Nim	0.33 ± 0.02^d^	96.60 ± 0.7^d^	>450^d^	1.53 ± 0.02^d^	>300

*Note*: IC_50_ value is the concentration required to produce 50% of inhibition of COX‐1 or COX‐2 for mean of three determinations using a COX‐1/COX‐2 assay kit. Nim: Nimesulide; selectivity index (SI) = IC_50_ COX‐1/IC_50_ COX‐2. For each column, means with the same letter are not significantly different for each other (*p >* 0.05).

Overall, these data suggest (and confirm) that the anti‐inflammatory properties of these EVOOEs are exerted through mechanisms involving several molecular targets and biochemical pathways and therefore go well beyond the simple COX inhibition.

## 3 Discussion

### 3.1 Quality Parameters of EVOOs

#### 3.1.1 Physicochemical Quality

Some olive cultivars are little known both most EVOO producers and food science experts. The Verdello belongs to the group of lesser‐known cultivars, mainly due to its low oil yield (about 20%) and the difficulty in cultivating and harvesting the fruit. Nowadays, Verdello olive trees have become rare and are found only in the area around the city of Messina. Nevertheless, EVOOs obtained from Verdello olives are highly prized, characterized by low acidity, high stability, and a persistent green sensory profile.

Nocellara del Belice, likely one of the most widespread native Sicilian varieties, is typical of the provinces of Agrigento and Trapani. With its large‐sized olives and adaptable nature, it produces both delicious table olives and high‐quality EVOO, thanks to its low acidity and high polyphenolic content. The Ogliarola Messinese olive tree, native to the provinces of Palermo and Messina, is distinguished by its dual‐purpose fruits, which is used both for fresh consumption and for the production of an exceptional EVOO, known for its balanced flavor.

Analysis of the qualitative characteristics of the olive oils obtained from these Sicilian cultivars revealed that all samples had free acidity levels below or very close to the limit established for EVOO [12].

The fatty acid methyl ester (FAME) profile of authentic EVOO is well‐defined and serves as a reliable marker of its purity [12]. Any deviation from this established profile may indicate adulteration with low‐cost oils or the presence of refined olive oil. Consequently, FAME analysis is a valuable tool for detecting the blending of EVOO with refined or non‐olive vegetable oils, as such adulteration results in characteristic changes in the FAME composition.

Notably, the Verdello and Ogliarola Messinese cultivars showed slightly elevated levels of free acidity (0.82% and 0.79%, respectively). These slight increases in free acidity can be attributed to various factors, including the harvesting method, storage temperature, and storage duration, as previously discussed by Famiani et al. and Taiti et al. [15, 16]. The oxidative status of an olive oil is of primary importance as it affects sensory, nutritional, organoleptic, and shelf‐life characteristics [17]. Oxidation leads to the formation of off‐flavors, such as those caused by carbonyl compounds and hydroperoxides, which reduce shelf‐life and consumer acceptance. Furthermore, the byproducts of oxidation have noxious effects on human health. All the quality parameters measured in this study, as required by EU Commission Regulations, highlight potential weaknesses in the olive oil production chain. In addition to tree cultivar and growing conditions, numerous other factors contribute to the quality of the oil. For instance, contamination by microorganisms (e.g., yeasts) can lead to an increase in all the parameters discussed here.

The values obtained for primary and secondary oxidation fell within the acceptable limits for EVOO in all three cultivars. The greatest oxidative stability was observed in Nocellara, with the lowest values recorded for peroxides, K232, and K270. Although the Verdello samples fell well within the quality limits set for EVOOs, they showed the highest levels of both primary and secondary oxidation. More importantly, the Δ*K* values confirm the genuineness and absence of adulteration in the entire series of samples.

#### 3.1.2 Fatty Acids

Determining the fatty acid composition of olive oil varieties (in this case from Nocellara del Belice, Ogliarola Messinese, and Verdello) provides essential information on their nutritional quality, oxidative stability, shelf‐life, suitability for culinary use, and potential flavor characteristics. Oleic acid is the most abundant fatty acid in olive oils. It is considered a key bioactive compound, responsible for the oil's health benefits and stability. The highest levels of oleic acid were found in the Nocellara del Belice samples (74.01%); slightly lower levels were found in the Verdello samples (72.75%) and to follow in the Ogliarola Messinese samples (69.72%). These oleic acid content values are significantly higher than those typically found in olive oils derived from other *Olea Europea* L. cultivars grown in the southern Mediterranean basin, where the average almost never reaches 70%. For example, in the study by Dabbou et al. [18], conducted on two wild varieties (*Oleasters K* and *M*) and two cultivated varieties (*Chemlali Sfax* and *Neb Jmel*) of *O. Europea* L. from the coastal region of Tunisia, the oleic acid content in the oils was 66.27%–63.18% and 60.64%–66.92%, respectively. A more recent study conducted by Affes et al. on two rare and underexplored olive cultivars, namely *Chemchali Gafsa* and *Fougi*, grown in southern Tunisia, revealed oleic acid values of 62.52% and 68.43%, respectively [19]. As for saturated fatty acids, palmitic acid was found to be the predominant component in all samples (range 10.79%–13.91%). This finding, in itself, is widely consistent with other studies. However, the average palmitic acid content in the oils of Tunisia cultivars is generally higher (e.g., 13.38%–16.39% in Dabbou et al. [18] and 11.90%–17.25% in Affes et al. [19]. In our study, since the fraction of saturated fatty acids is higher in Ogliarola, samples of this cultivar can be considered potentially more resistant to oxidation compared to those of Nocellara and Verdello. As mentioned above, linoleic acid was the third most abundant fatty acid in all samples (range 9.48%–10.81%). The order in terms of fatty acid content “oleic acid >> palmitic acid > linoleic acid” is sometimes reversed in the last two as regards olive oils from Tunisian cultivars, as in the case of *Fougi* (11.90% vs. 14.55%) [19], and *Chemlali Sfax* (15.45% vs. 17.14%) [18]. Furthermore, the values determined for specific fatty acids, as regulated by the EU Commission, were found to comply with the requirements [12]. It is worth mentioning the recognized beneficial role of PUFAs on health [20].

#### 3.1.3 Phenolic Composition of EVOOEs

Regarding the polyphenolic composition of EVOOs, the Nocellara variety showed the richest polyphenolic profile, particularly in terms of OHT (5.23 µg mL^−1^), OAC (65.20 µg mL^−1^), and OCA (195.56 µg mL^−1^), suggesting greater health benefits. The Verdello variety, in comparison, had slightly lower levels of OHT (3.82 µg mL^−1^), and OCA (183.21 µg mL^−1^), and moderate levels of OAC (27.33 µg mL^−1^), while the Ogliarola variety had lower levels of all identified polyphenolic compounds, including TYR (see Table 4). As in other studies on EVOOs obtained from olive trees in the Mediterranean basin, secoiridoids are the most abundant polyphenolic component, although the qualitative and quantitative composition is rather difficult to compare. For instance, olive oils from autochthonous and introduced cultivars in Tunisia are particularly rich in OEP derivatives (Loubiri 2017). These differences likely reflect both genetic and environmental factors that influence the biosynthesis of polyphenols in the respective cultivars [21]. The content of polyphenols found in our samples is somehow in compliance with the European Food Safety Authority (EFSA) claim for EVOO health benefits, that indicates ∼200 mg kg^−1^ of TYR and derivatives in the oil. The values obtained for OCA and OAC are in accordance with previous findings for EVOOs of medium‐high quality [22]. The lower values of TYR and OHT found are justified by the fact that they are breakdown products of OAC and OCA, the main polyphenols typical of young EVOOs. Variability in OAC content was observed among samples, seemingly attributable to the method used for the production of EVOO. Indeed, it has been demonstrated that the stage of maturation, the cultivar, and the time of harvest are crucial factors, particularly for the OAC content in EVOO [23, 24].

#### 3.1.4 Antioxidant Capacity

Regarding the antioxidant activity of the three EVOOEs, the results showed that Verdello has antioxidant capacity comparable to that of Nocellara, a variety widely recognized for producing high‐quality oil rich in bioactive compounds endowed with radical scavenging properties [25]. This outcome highlights Verdello as a promising yet underexplored cultivar with notable nutraceutical potential. Ogliarola, on the other hand, displayed reduced activity compared to the other two EVOOEs.

The decrease in antioxidant capacity mirrored the trend observed for total phenolic compounds. Phenolic compounds are widely recognized as the main antioxidants present in olive extracts [26]. The overall antioxidant capacity of an extract encompasses both its “antiradical” and “antioxidant” activities, which do not always coincide. To gain a more comprehensive understanding of the antioxidant capacity of the extracts, four different assays (DPPH, ABTS, ORAC, and FRAP) were employed, each targeting distinct antioxidant defense mechanisms. Antiradical activity refers to the ability of compounds to neutralize free radicals, as assessed by assays such as ABTS and DPPH. In contrast, antioxidant activity reflects the capacity to prevent oxidative processes, as measured by assays like FRAP and ORAC [13].

Consistent with the findings of Samaniego Sánchez et al. [27], a positive correlation (Table 6) was observed between the mainly compounds present in the extracts and the antioxidant capacity, particularly in the DPPH and ABTS tests [27]. However, this correlation was less evident, for some compounds (OCA and OAC) except for ORAC test, suggesting that additional factors, such as the specific chemical structure of individual molecules, their interaction with other oil constituents, or differing assay mechanisms, may influence the antioxidant outcomes.

These findings underline the complex nature of antioxidant behavior in natural matrices like EVOO and reinforce the importance of employing multiple, complementary in vitro assays to gain a comprehensive understanding of antioxidant capacity. Such an approach is especially valuable when characterizing less‐studied cultivars like Verdello, which may hold untapped potential for functional food and nutraceutical development.

#### 3.1.5 Anti‐Inflammatory Properties

Beyond its well‐established antioxidant properties EVOO is widely recognized for its potent anti‐inflammatory activity, largely attributed to its rich content of phenolic compounds such as OHT, OCA, and OAC These bioactive molecules modulate inflammatory processes by interfering with key signaling pathways, reducing the production of pro‐inflammatory cytokines, and regulating enzymes (including COX) involved in the synthesis of eicosanoids [28]. Through these mechanisms, EVOO contributes to the attenuation of chronic inflammation, a condition closely linked to metabolic, cardiovascular, and degenerative diseases [29, 31, 32].

In this context, the present analysis of the three different cultivars provides further insight into the inhibitory effects of EVOOEs on COX enzymes. Olive oil phenolic compounds, particularly oleocanthal, are known to inhibit both COX‐1 and COX‐2 activity, mimicking the mechanism of action of NSAIDs, while also reducing lipopolysaccharide‐induced expression of inflammatory mediators such as TNF‐α, IL‐6, and IL‐1β. Similarly, OAC has been shown to reduce nitric oxide production and inhibit enzymes involved in arachidonic acid metabolism [29].

Our findings are consistent with previous reports confirming the inhibitory potential of EVOOEs on both COX isoforms [30, 31]. The IC_50_ values obtained fall within the range typically reported for crude plant extracts, which generally exhibit enzyme inhibition in the tens to hundreds of µg/mL, reflecting their complex chemical composition compared to purified compounds [32, 33]. In this test, the inhibitory profile exerted by our three EVOOEs was clear: while COX‐1 inhibition remained relatively modest (21.60%–25.39%; see Table 7), COX‐2 inhibition was significantly more pronounced. Specifically, Verdello (61.20%) and Nocellara (57.08%) exhibited the strongest inhibitory activity, followed by Ogliarola (43.70%). This selective inhibitory profile is particularly relevant from a therapeutic perspective, as greater selectivity toward COX‐2 could offer anti‐inflammatory benefits while reducing potential gastrointestinal side effects. This trend is further supported by the selectivity index (SI), calculated as the ratio of IC_50_ for COX‐1 to COX‐2. Both Nocellara (SI = 2.6) and Verdello (SI = 2.4) demonstrated a clear preference for COX‐2 inhibition. The observed differences among cultivars may be attributed to variations in their phenolic composition. In particular, higher levels of secoiridoids such as OCA, OHT, and related derivatives in Nocellara and Verdello likely contribute to their enhanced COX‐2 inhibitory activity. Among these, OCA appears to play a central role, given its well‐documented ability to inhibit COX enzymes through mechanisms comparable to synthetic COX‐2 inhibitors.

The cultivar‐dependent differences observed in this study further suggest that the health benefits of EVOO may vary according to its phenolic profile, with certain varieties offering enhanced anti‐inflammatory properties and improved therapeutic potential [29].

## 4 Conclusions

This study evaluated the qualitative and bioactive properties of EVOOEs from three Sicilian cultivars that is Nocellara del Belice, Ogliarola Messinese, and the newly characterized Verdello. The results revealed that the cultivars, except for Ogliarola Messinese that slightly exceeds the free acidity threshold, largely met the established quality criteria for EVOO. In particular, EVOOEs of Nocellara del Belice and Verdello exhibited superior levels of total polyphenols and flavonoid compounds, which were strongly correlated with enhanced antioxidant activity and consistent with previous findings linking polyphenolic content to oxidative stability and health‐promoting effects [14, 34].

The anti‐inflammatory activity assessed via COX‐1 and COX‐2 inhibition revealed a pattern of moderate COX‐1 and more pronounced COX‐2 inhibition across all cultivars. This selective COX‐2 inhibition is particularly important, as it suggests potential health benefits with fewer gastrointestinal side effects commonly associated with non‐selective COX inhibition [30, 35]. The polyphenolic profile, particularly the presence of OCA, OHT, and secoiridoids, likely contributes to these effects, with Nocellara and Verdello being especially rich in these bioactive compounds.

Verdello, previously uncharacterized, has consistently shown good results in all parameters examined, positioning itself as a cultivar of high agronomic and nutraceutical value. These findings provide a compelling case for the increased cultivation and production of EVOO from Verdello, supporting its preservation as a local variety and its potential as a marketable, high‐quality olive oil. In contrast, Ogliarola exhibited comparatively lower phenolic content and antioxidant capacity, suggesting a need for further agronomic and/or technological optimization to improve its bioactive profile.

Overall, our findings reinforce the role of EVOO, particularly from selected cultivars such as Nocellara del Belice and Verdello, as a functional food with meaningful antioxidant and anti‐inflammatory properties. These properties not only support general health and disease prevention but also point to EVOO's potential as a complementary dietary strategy in managing chronic inflammatory conditions. Furthermore, the study emphasizes the importance of preserving local biodiversity and promoting underutilized cultivars to enhance both the nutritional quality and cultural heritage of olive oil production in regions like Sicily.

## 5 Experimental Section

### 5.1 Materials and Methods

#### 5.1.1 Chemicals and Reagents

All solvents, Folin–Ciocalteu reagent, sodium carbonate (Na_2_CO_3_), 2,2‐diphenyl,1‐picrylhydrazyl (DPPH), 2,4,6‐tri(2‐pyridyl)‐1,3,5‐triazine (TPTZ), ferric chloride (FeCl_3_·6H_2_O), gallic acid, acetic acid, ferric sulfate (FeSO_4_·7H_2_O), sodium acetate, 2,2’‐azino‐bis(3‐ethylbenzothiazoline‐6‐sulphonic acid) (ABTS), potassium persulfate, phosphate‐buffered saline (PBS) and Trolox were purchased from Sigma‐Aldrich (St. Louis, MO, USA). All polyphenolic reference standards were supplied by Sigma‐Aldrich‐Merck (Darmstadt, Germany) and were: oleuropein (OEP), vanillic acid (VAN), *p*‐coumaric acid (COU), tyrosol (TYR), hydroxytyrosol (OHT), oleacein (OAC), oleocanthal (OCA), syringic acid (SYR).

### 5.2 Extra Virgin Olive Oils (EVOOs)

**FIGURE 5:**
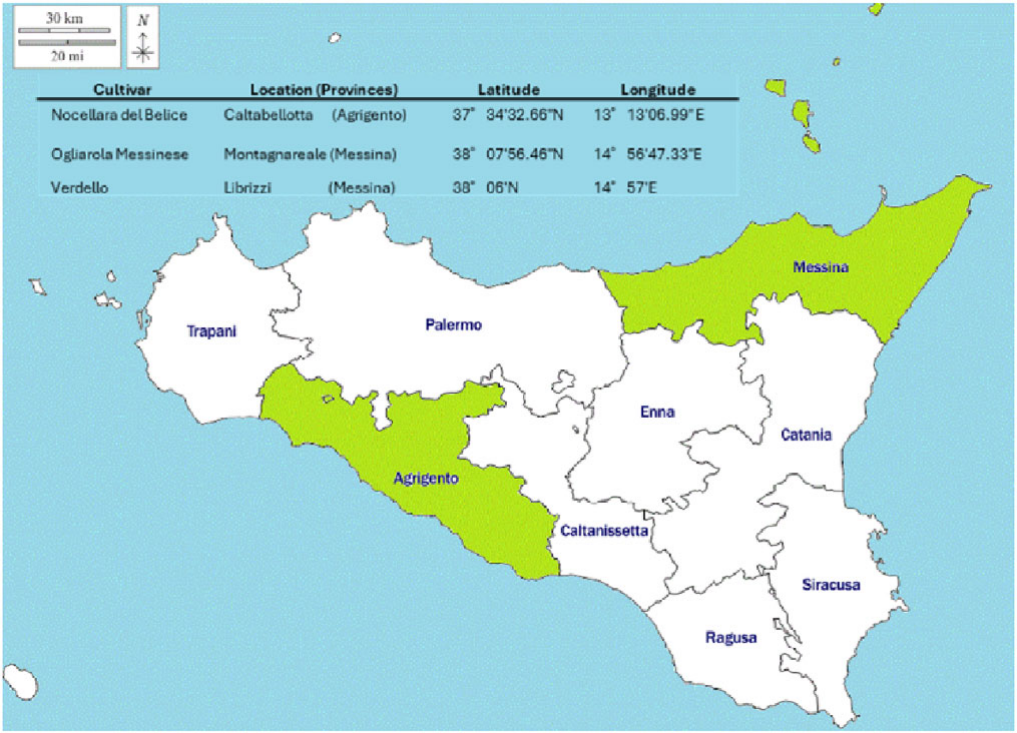
Cultivar provinces of origin (in green), with geographic references for the olive harvesting locations (table). The initial version was generated using Gemini (Google; accessed March 2026) and subsequently refined by the authors using Microsoft PowerPoint.

This study used olive oils from three ancient cultivars of *O. europaea* L., namely Nocellara del Belice, Ogliarola Messinese, and Verdello, grown in Sicily. Olives were carefully harvested in November 2022 in two different cultivation areas located in the Messina (Ogliarola Messinese and Verdello) and Agrigento (Nocellara del Belice) provinces (Figure 5) (Sicily, Italy).

Olive oil extraction was performed using Alfa Laval technology (Alfa Laval, Italy) through a continuous centrifugal process. After washing, olives were crushed with a disc crusher and the resulting paste was malaxed under controlled temperature using heat exchangers that heat the paste quickly and precisely, optimizing yield without damaging the polyphenols. Oil was separated by a three‐phase horizontal decanter centrifuge (oil, vegetation water, and pomace), followed by final purification using a vertical centrifugal disc separator to remove residual water and impurities. This system ensured high hygiene standards and preservation of oil quality.

Three different bottles of 500 mL of each cultivar were kept at constant temperature of 4°C until analysis using amber flasks.

### 5.3 Conventional Assays on EVOOs

#### 5.3.1 Determination of Physicochemical Quality

The parameters of regulated physicochemical quality (free acidity, peroxide index and absorbance in the ultraviolet, K232 and K270) were determined following the analytical methods described in Regulation (EC) no/2568/91 of the Commission of the European Union [12].

Free acidity was determined by means of titration: 0.1 M NaOH as titrant was added to a solution containing 5 g of EVOO, 90 mL of ethanol/diethyl ether (1:2, v/v), phenolphthalein as indicator.

Peroxides were assessed through the following procedure: 1 g of oil was dispersed in 25 mL of an acetic acid/chloroform (3:2, v/v) solution, added with 0.5 mL of a potassium iodide saturated solution. The mixture was kept in the dark for 5 min, then added with 75 mL of distilled water, and finally titrated with 0.01 N sodium thiosulfate, by using starch paste as indicator. The results were expressed as mEq of O_2_ kg^−1^ oil.

The oxidation parameters, namely K232, K270, and Δ*K*, were spectrophotometrically determined by means of a UV–vis spectrophotometer by Shimadzu, model UV‐2401 PC (Shimadzu Italia, Milan, Italy). Prior to analysis, each oil sample was dissolved in cyclohexane (0.01%, w/v). Absorbance was measured at 232 and 270 nm wavelengths, for K232 and K270, respectively. The value of Δ*K* was obtained by applying the equation:

$$\Delta K=K270-\left[\left(K264+K276\right)/2\right]$$

#### 5.3.2 Gas Chromatographic Analysis of FAMEs

Prior to analysis, oil samples underwent transesterification with methanolic solution of potassium hydroxide at room temperature [36].

The acidic profile of EVOOs was thereafter determined by means of a GC system provided by DANI (Milan, Italy), equipped with a split/splitless injector (split ratio, 1:50; sample volume, 1 µL) and an FID detector. The capillary column was a Phenomenex ZB‐wax, 30 m × 0.25 mm × 0.25 µm. The oven was temperature programmed as follows: 50°C (2 min) to 210°C (15 min) at 3°C min^−1^. Injection and detection temperatures were both set at 240°C. Carrier gas (He) linear velocity was 30 cm s^−1^. The peak assignment took place by comparing the unknown retention times to those of a FAMEs mixture provided by Merck (Supelco 37 Component FAME Mix). Quantitative analysis was based on peak area percentage method.

#### 5.3.3 Extra Virgin Olive Oil Extracts (EVOOEs)

The phenolic hydroalcoholic extracts preparation followed the procedure reported by the International Olive Council (COI) with slight modifications [37, 38]. The oil samples (3.5 g) were added to 8.75 mL of a methanol/water (80:20, v/v) mixture; after 3–5 min of vigorous stirring, the mix was centrifuged for 25 min (4000 rpm at 25°C). The hydroalcoholic phase was collected using a separation funnel. After that, another 8.75 mL of methanol/water (80:20) was added to the same olive oil and again centrifuged for 25 min. The hydroalcoholic phase was collected and added to the same separation funnel. A volume of 7 mL of hexane was added to the separation funnel, mixed for 3–5 min, and let to stand for 2 min. After easily seeing the separation between phases, the methanol/water phase was collected and transferred to a round‐bottom flask. The latter was taken to the rotary evaporator (37°C) to eliminate methanol and water. The dry EVOOEs were dissolved in methanol, ready for analyses.

The extraction yields of the samples were calculated based on the weight of the residues obtained after extraction with solvents and the vacuum concentration of the filtrates. The highest value was recorded for Ogliarola (16.6%) followed by Nocellara (14.8%) and Verdello (13.2%).

### 5.4 Characterization of Polyphenols in EVOOEs

#### 5.4.1 Total Polyphenolic Content

The total phenolic content of the EVOOEs was determined by the Folin–Ciocalteu colorimetric assay [39]. Initially, gallic acid (standard) and samples were prepared at various concentrations in methanol (gallic acid 0–300 µg mL^−1^; EVOOEs 0–2 mg mL^−1^). Subsequently, 50 µL of each sample were taken and diluted to 500 µL with water in borosilicate tubes. Following this, 500 µL of Folin–Ciocalteu reagent was added to each dilution and vigorously shaken. After a 3 min incubation period, 500 µL of freshly prepared 10% Na_2_CO_3_ was added. The mixture was then incubated in the dark for 1 h, shaking every 10 min. Finally, the solution was transferred to cuvettes, and the absorbance was read at 786 nm against methanol using a UV–vis spectrophotometer.

#### 5.4.2 Total Flavonoid Content

The total flavonoid content was determined as previously described [40]. Rutin (standard) and samples were solubilized in methanol prepared at various concentrations (0–1000 µg mL^−1^). Subsequently, 50 µL of each sample was taken and diluted to 500 µL with water in borosilicate tubes. Following this, 30 µL of 5% NaNO_2_ was added to the tubes, and after a 5 min incubation period, 60 µL of 10% AlCl_3_ were added. After a further 6‐min wait, 200 µL of 1 M NaOH and 210 µL of water were added. The solution was vigorously shaken, transferred to cuvettes, and the absorbance was read at 510 nm against methanol using the UV–vis spectrophotometer.

#### 5.4.3 HPLC Analysis

The EVOOEs underwent HPLC analysis for the elucidation and quantification of the polyphenolic fraction. Prior to injection, the dried extracts were reconstituted with 2.5 mL of methanol, added with a fixed amount of syringic acid (25 µL of a 50 ppm solution), and filtered through syringe filters. Analyses were carried out on a Shimadzu Prominence LC‐20A system (Shimadzu, Kyoto, Japan) equipped with LC‐20AD feeding solvent system, DGU‐20A3R degasser, CTO‐20A column oven, SPD‐M20A diode array detector, according to previously published methodology [41].

The separation of analytes took place on an Ascentis C18 column, 25 cm × 4.6 mm × 5 µm, provided by Supelco‐Merck (Darmstadt, Germany). The binary solvent system was composed of water/formic acid, 99.7:0.3 v/v (Solvent A); acetonitrile/formic acid, 99.7:0.3 v/v (Solvent B). The gradient elution program was as follows: 0 min, 2% B; 7 min, 7% B; 60 min, 60% B; 75 min, 100% B. The flow rate was 1 mL/min, injection volume was 20 µL. PDA acquisition was set in the wavelength range 200–500 nm and chromatograms were extracted at 280 nm. Data were handled by means of LabSolution software. A standard mixture of OEP, VAN, TYR, OHT, OAC, and OCA in methanol was prepared at a concentration of 100 µg mL^−1^ for qualitative analysis and method validation assessment (retention time repeatability). Single solutions of standard polyphenols (TYR, OHT, OAC, and OCA) were prepared at six different concentrations, namely 250–125–62.5–31.2–10.0–1.0 mg mL^−1^, in order to build the linear regression model through the calibration curve. Each concentration level was injected in triplicate. As for real samples, each standard solution was added with syringic acid as internal standard (25 µL of a 50 ppm solution).

#### 5.4.4 Antioxidant Capacity Tests

##### 5.4.4.1 2,2‐Diphenyl‐1‐picrylhydrazyl Test

The free‐radical‐scavenging capacity of the EVOOEs was determined by the 2,2‐diphenyl‐1‐picrylhydrazyl (DPPH) assay [42]. Trolox (standard) and EVOOEs were initially prepared at various concentrations (Trolox range 0–1 mM; Nocellara and Verdello range 0–2 mg mL^−1^ and Ogliarola 0–3 mg mL^−1^).

The right amount of DPPH was dissolved in methanol and sonicated in an ultra‐sonic bath until complete solubility was achieved. Prior to use, the absorbance of DPPH was verified, ensuring it fell within the range of 0.90 ± 0.02; if necessary, adjustments were made by diluting it with methanol and re‐measuring until the desired absorbance was attained. In tubes, 37.5 µL of samples at various concentrations were added to 1.5 mL of DPPH and agitated using a vortex mixer. The tubes were then placed in darkness for a 20‐min reaction period. Subsequently, the solutions were transferred to cuvettes, and the absorbance was measured at 517 nm against methanol.

##### 5.4.4.2 2,2’‐Azino‐bis‐(3‐ethylbenzothiazoline‐6‐sulfonic acid) Assay

This method determines the capacity of the EVOOEs to quench the azino‐bis‐(3‐ethylbenzothiazoline‐6‐sulfonic acid) radical (ABTS ·+). In our experiments, according with Chelly et al. [43] the ABTS ·+ radical cation was produced by the oxidation of 1.7 mM ABTS with potassium persulfate (4.3 mM final concentration) in water. The mixture was allowed to stand in the dark at room temperature for 12–16 h before use, and then the ABTS ^·+^ solution was diluted with phosphate buffered saline (PBS) at pH 7.4 to give an absorbance of 0.7 ± 0.02 at 734 nm. Trolox and samples were then prepared at various concentrations (Trolox range 0.016–0.25 mM; Ogliarola range 0–2 mg mL^−1^; and Nocellara and Verdello range 0.125–2 mg mL^−1^) and 100 µL of each sample to be tested or the vehicle alone (MeOH) was added to 2 mL of the ABTS ^·+^ solution, and the absorbance was recorded at 734 nm in a UV–vis spectrophotometer after allowing the reaction to stand for 6 min in the dark at room temperature. Trolox was used as blank. Each determination was carried out in triplicate.

##### 5.4.4.3 Ferric‐Reducing/Antioxidant Power Assay

The ferric‐reducing/antioxidant power (FRAP) of the phenolic fraction extracts was evaluated as previously described [44]. FeSO_4_ and samples were prepared at various concentrations (FeSO_4_ range 0–1 mM; EVOOEs range 0–2 mg mL^−1^). Next, the FRAP reagent was prepared, consisting of acetate buffer (10 V), TPTZ (1 V), and FeCl_3_·6H_2_O (1 V) in a 10:1:1 volume ratio, respectively, with a pH of 3.6. The acetate buffer was prepared by mixing 3.1 g of sodium acetate 3·H_2_O with 16 mL of acetic acid per 1 L of buffer. Additionally, a 20 mM FeCl_3_·6H_2_O solution was prepared in water, and 10 mM TPTZ was prepared by diluting 40 mM HCl with water and then mixing it with TPTZ, which was subsequently sonicated. After the FRAP reagent was prepared, 1 mL was added to tubes, followed by 50 µL of samples, and the mixture was allowed to incubate for 4 min after agitation. Finally, the absorbance was measured at 593 nm.

##### 5.4.4.4 Oxygen Radical Absorbance Capacity Assay

The protocol for the Oxygen Radical Absorbance Capacity with Fluorescein (ORAC‐FL) assay was evaluated as previously described [45]. The assay started with the solubilization of sodium hydrogen phosphate (Na_2_HPO_4_) and potassium dihydrogen phosphate (KH_2_PO_4_) at concentrations of 75 mM each in deionized water. A phosphate buffer solution at 75 mM with a pH of 7.4 was then prepared by combining 81.8 mL of the Na_2_HPO_4_ solution with 18.2 mL of the KH_2_PO_4_ solution. Fluorescein was subsequently solubilized in the phosphate buffer solution at a concentration of 117 nM. 2,2'‐Azobis(2‐amidinopropane)dihydrochloride (AAPH) was dissolved in the phosphate buffer solution at a concentration of 40 mM and kept on ice until use. Standard solutions and samples at various concentrations were prepared (Trolox used as a standard in the range 0–75 µM; Nocellara and Verdello used in the range 0–10 µg mL^−1^; Ogliarola used in the range 0–25 µg mL^−1^), with the final dilutions solubilized in the phosphate buffer solution after an initial solubilization in methanol. Fluorescence measurements were conducted using a spectrofluorophotometer RF‐5301 PC (Shimadzu, Japan) with excitation and emission wavelengths set at 485 and 520 nm, respectively. In cuvettes, 20 µL of the standard or sample solution was placed, followed by the addition of 120 µL of fluorescein solution. The solution mixture was thoroughly mixed by pipette and then placed inside the spectrofluorophotometer, maintained at 37°C. After a 15‐min incubation period, 60 µL of AAPH solution were added to the cuvette, and the solution mixture was gently agitated using the pipette tip. Fluorescence measurements were recorded at 1.5 min intervals for 60 min.

### 5.5 In Vitro COX‐1 and COX‐2 Inhibition Assay

The inhibitory potency of EVOOEs for COX‐1 and COX‐2 was established using the COX (human) Inhibitor Screening Assay kit (Item No. 701230, Cayman Chemical Company, Ann Arbor, MI, USA) according to the manufacturer's instructions. Briefly, background tubes (160 µL reaction buffer, 10 µL heme, and 10 µL inactive COX‐1 or COX‐2), COX 100% initial activity tubes (160 µL reaction buffer, 10 µL heme, and 10 µL COX‐1 or COX‐2), and COX inhibitor tubes (same content of 100% initial activity tubes) were set up. After setting up the tubes, 10 µL of investigated samples (range 10–60 µg mL^−1^) or reference standard (nimesulide range 1.5–9 µg mL^−1^) in DMSO were added to COX inhibitor tubes and 10 µL DMSO to the background and 100% initial activity tubes. After incubation for 10 min at 37°C, the COX reactions were initiated by adding arachidonic acid (10 µL) incubating for other 30 s at 37°C. Then 30 µL of saturated SnCl2 solution was added to stop enzyme reaction. The PGF_2α_ formed by COX reactions was quantified by enzyme immunoassay (EIA). The precoated with Ache Tracer (50 µL) and antiserum (50 µL) 96‐well plate containing compounds was incubated for 18 h at room temperature. Then the plate was washed to remove any unbound reagent and Ellman's reagent (200 µL) was added followed by incubation in the dark for 60 min at room temperature. The product PGF_2α_ was determinate by microplate spectrophotometer (iMark Microplate Absorbance Reader; BIO‐Rad Italy) at 415 nm. IC50 values were calculated from a concentration–inhibition response curve. The inhibition of COX‐1 and COX‐2 activities was measured by comparing the amount of PG produced in inhibitor tube with 100% activity tubes.

### 5.6 Statistical Analysis

All experiments were conducted in triplicate and repeated three times. Data are presented as mean ± standard deviation (SD) from three independent experiments. Statistical comparisons were performed using one‐way analysis of variance, followed by Tukey's Honest Significant Difference post‐hoc test, using the ezANOVA software (https://people.cas.sc.edu/rorden/ezanova/index.html). Differences between groups and treatments were considered statistically significant at *p* 0.05.

## Author Contributions

**Ali Mert Yetisgin**: conceptualization, methodology, formal analysis, investigation, writing – original draft preparation. **Giovanni Bartolomeo**: methodology, formal analysis, investigation. **Nicola Cicero**: data curation. **Nicola Micale**: writing – review and editing. **Rosaria Costa**: methodology, validation, investigation, data curation, writing – original draft preparation, project administration. **Mariateresa Cristani**: conceptualization, methodology, validation, investigation, resources, data curation, writing – original draft preparation, writing – review and editing, visualization, supervision, project administration, funding acquisition. All authors have read and agreed to the published version of the manuscript.

## Funding

This research received no external funding.

## Conflicts of Interest

The authors declare no conflicts of interest.

## Data Availability

The data that support the findings of this study are available from the corresponding author upon reasonable request.

## References

[1] A. J. P. O. De Almeida , J. C. P. L. De Oliveira , L. V. Da Silva Pontes , et al., “ROS: Basic Concepts, Sources, Cellular Signaling, and Its Implications in Aging Pathways,” Oxidative Medicine and Cellular Longevity 2022 (2022): 1–23. 10.1155/2022/1225578 PMC9605829 36312897

[2] S. K. Bardaweel , M. Gul , M. Alzweiri , A. Ishaqat , H. A. Al Salamat , and R. M. Bashatwah , “Reactive Oxygen Species: The Dual Role in Physiological and Pathological Conditions of the Human Body,” Eurasian Journal of Medicine 50 (2019): 193–201. 10.5152/eurasianjmed.2018.17397 PMC6263229 30515042

[3] T. A. F. Aguilar , B. C. H. Navarro , and J. A. M. Pérez , “Endogenous Antioxidants: A Review of their Role in Oxidative Stress,” in: A Master Regulator of Oxidative Stress – The Transcription Factor Nrf2. (InTech, 2016), 10.5772/65715.

[4] A. Rana , M. Samtiya , T. Dhewa , V. Mishra , and R. E. Aluko , “Health Benefits of Polyphenols: A Concise Review,” Journal of Food Biochemistry 46 (2022): e14264, 10.1111/jfbc.14264. 35694805

[5] A. Karković Marković , J. Torić , M. Barbarić , and C. J. Brala , “Hydroxytyrosol, Tyrosol and Derivatives and Their Potential Effects on Human Health,” Molecules 24 (2019): 2001. 31137753 10.3390/molecules24102001 PMC6571782

[6] G. Serreli and M. Deiana , “Extra Virgin Olive Oil Polyphenols: Modulation of Cellular Pathways Related to Oxidant Species and Inflammation in Aging,” Cells 9 (2020): 478. 32093046 10.3390/cells9020478 PMC7072812

[7] K.‐L. Pang and K.‐Y. Chin , “The Biological Activities of Oleocanthal From a Molecular Perspective,” Nutrients 10 (2018): 570. 29734791 10.3390/nu10050570 PMC5986450

[8] M. González‐Rodríguez , D. Ait Edjoudi , A. Cordero‐Barreal , et al., “Oleocanthal, an Antioxidant Phenolic Compound in Extra Virgin Olive Oil (EVOO): A Comprehensive Systematic Review of Its Potential in Inflammation and Cancer,” Antioxidants 12 (2023): 2112. 38136231 10.3390/antiox12122112 PMC10741130

[9] R. Costa , G. Bartolomeo , E. Saija , R. Rando , A. Albergamo , and G. Dugo , “Determination of Alkyl Esters Content in PDO Extra Virgin Olive Oils From Sicily,” Journal of Food Quality 2017 (2017): 1–7.

[10] D. De Santis , S. Ferri , G. Milana , G. Turchetti , and M. Modesti , “Stability of Monovarietal Sicilian Olive Oils Under Different Storage Condition: Chemical Composition, Sensory Characteristics, and Consumer Preference,” Heliyon 10 (2024): e29833. 38699013 10.1016/j.heliyon.2024.e29833 PMC11064150

[11] P. Agozzino , G. Avellone , D. Bongiorno , et al., “Determination of the Cultivar and Aging of Sicilian Olive Oils Using HPLC‐MS and Linear Discriminant Analysis,” Journal of Mass Spectrometry 45 (2010): 989–995. 20821559 10.1002/jms.1791

[12] COMMISSION REGULATION (EEC) N° 2568/91, Regulation ‐ 2568/91 ‐ EN—EUR‐Lex, accessed August 20, 2025, https://eur‐lex.europa.eu/eli/reg/1991/2568/oj/eng.

[13] F. Brahmi , B. Mechri , M. Dhibi , and M. Hammami , “Variations in Phenolic Compounds and Antiradical Scavenging Activity of *Olea europaea* Leaves and Fruits Extracts Collected in Two Different Seasons,” Industrial Crops and Products 49 (2013): 256–264.

[14] M. Servili , B. Sordini , S. Esposto , et al., “Biological Activities of Phenolic Compounds of Extra Virgin Olive Oil,” Antioxidants 3 (2013): 1–23. 26784660 10.3390/antiox3010001 PMC4665453

[15] F. Famiani , D. Farinelli , S. Urbani , et al., “Harvesting System and Fruit Storage Affect Basic Quality Parameters and Phenolic and Volatile Compounds of Oils From Intensive and Super‐Intensive Olive Orchards,” Scientia Horticulturae 263 (2020): 109045.

[16] C. Taiti , E. Masi , F. Flamminii , C. Di Mattia , S. Mancuso , and E. Marone , “Does the Harvest Type Affect Olive Health? Influence of the Harvesting System and Storage Time on the Chemical, Volatile and Sensory Qualities of Extra Virgin Olive Oils,” Plants 12 (2023): 3843. 38005740 10.3390/plants12223843 PMC10674536

[17] M. Vendrell Calatayud , X. Li , S. Brizzolara , P. Tonutti , and S. C. Wang , “Storage Effect on Olive Oil Phenols: Cultivar‐Specific Responses,” Frontiers in Nutrition 11 (2024): 1382551. 39077155 10.3389/fnut.2024.1382551 PMC11285335

[18] S. Dabbou , S. Dabbou , R. Selvaggini , et al., “Comparison of the Chemical Composition and the Organoleptic Profile of Virgin Olive Oil From Two Wild and Two Cultivated Tunisian *Olea europaea* ,” Chemistry and Biodiversity 8 (2011): 189–202. 21259429 10.1002/cbdv.201000086

[19] M. Affes , D. Dallali , H. Jabeur , et al., “A Comparative Phytochemical Analysis of Olive Oils Derived From Two Underexplored Tunisian Cultivars: Chemchali and Fougi,” Chemistry and Biodiversity 22 (2025): e00574. 40609026 10.1002/cbdv.202500574 PMC12629151

[20] R. Costa , M. Beccaria , E. Grasso , et al., “Sample Preparation Techniques Coupled to Advanced Chromatographic Methods for Marine Organisms Investigation,” Analytica Chimica Acta 875 (2015): 41–53. 25937105 10.1016/j.aca.2015.03.032

[21] M. L. Clodoveo , S. Camposeo , R. Amirante , G. Dugo , N. Cicero , and D. Boskou , Olive and Olive Oil Bioactive Constituents (Elsevier, 2015), 179–215.

[22] J. E. Salsano , M. Digiacomo , D. Cuffaro , S. Bertini , and M. Macchia , “Content Variations in Oleocanthalic Acid and Other Phenolic Compounds in Extra‐Virgin Olive Oil During Storage,” Foods 11 (2022): 1354. 35564077 10.3390/foods11091354 PMC9105779

[23] S. Charoenprasert and A. Mitchell , “Factors Influencing Phenolic Compounds in Table Olives (*Olea europaea*),” Journal of Agricultural and Food Chemistry 60 (2012): 7081–7095. 22720792 10.1021/jf3017699

[24] G. Sivakumar , C. Briccoli Bati , and N. Uccella , “HPLC‐MS Screening of the Antioxidant Profile of Italian Olive Cultivars,” Chemistry of Natural Compounds 41 (2005): 588–591.

[25] C. Negro , A. Aprile , A. Luvisi , et al., “Phenolic Profile and Antioxidant Activity of Italian Monovarietal Extra Virgin Olive Oils,” Antioxidants 8 (2019): 161. 31195713 10.3390/antiox8060161 PMC6617199

[26] A. Sousa , R. Malheiro , S. Casal , A. Bento , and J. A. Pereira , “Antioxidant Activity and Phenolic Composition of Cv. Cobrançosa Olives Affected Through the Maturation Process,” Journal of Functional Foods 11 (2014): 20–29.

[27] C. Samaniego Sánchez , A. M. Troncoso González , M. C. García‐Parrilla , J. J. Quesada Granados , H. López García De La Serrana , and M. C. López Martínez , “Different Radical Scavenging Tests in Virgin Olive Oil and Their Relation to the Total Phenol Content,” Analytica Chimica Acta 593 (2007): 103–107. 17531830 10.1016/j.aca.2007.04.037

[28] K. Agrawal , E. Melliou , X. Li , et al., “Oleocanthal‐Rich Extra virgin Olive Oil Demonstrates Acute Anti‐Platelet Effects in Healthy Men in a Randomized Trial,” Journal of Functional Foods 36 (2017): 84–93. 29904393 10.1016/j.jff.2017.06.046 PMC5995573

[29] V. Costa , M. Costa , R. A. Videira , P. B. Andrade , and F. Paiva‐Martins , “Anti‐Inflammatory Activity of Olive Oil Polyphenols—The Role of Oleacein and Its Metabolites,” Biomedicines 10 (2022): 2990. 36428559 10.3390/biomedicines10112990 PMC9687571

[30] G. K. Beauchamp , R. S. J. Keast , D. Morel , et al., “Ibuprofen‐Like Activity in Extra‐Virgin Olive Oil,” Nature 437 (2005): 45–46. 16136122 10.1038/437045a

[31] R. Estruch , E. Ros , J. Salas‐Salvadó , et al., “Primary Prevention of Cardiovascular Disease With a Mediterranean Diet Supplemented With Extra‐Virgin Olive Oil or Nuts,” New England Journal of Medicine 378 (2018): e34, 10.1056/NEJMoa1800389. 29897866

[32] J. L. S. Taylor and J. Van Staden , “COX‐1 Inhibitory Activity in Extracts From Eucomis L'Herit. Species,” Journal of Ethnopharmacology 75 (2001): 257–265. 11297860 10.1016/s0378-8741(01)00205-7

[33] G. G. Ambati and S. M. Jachak , “Natural Product Inhibitors of Cyclooxygenase (COX) Enzyme: A Review on Current Status and Future Perspectives,” Current Medicinal Chemistry 28 (2021): 1877–1905. 32484764 10.2174/0929867327666200602131100

[34] K. L. Tuck and P. J. Hayball , “Major Phenolic Compounds in Olive Oil: Metabolism and Health Effects,” Journal of Nutritional Biochemistry 13 (2002): 636–644. 12550060 10.1016/s0955-2863(02)00229-2

[35] J. R. Vane and R. M. Botting , “Mechanism of Action of Antiinflammatory Drugs,” International Journal of Tissue Reactions 20 (1998): 3–15. 9561441

[36] https://www.internationaloliveoil.org/wp‐content/uploads/2019/11/COI‐T.20‐Doc.‐No‐33‐Rev.‐1‐2017.pdf.

[37] C. D. Goldsmith , Fate of the Paorlic Compounds During Olive Oil Production With the Traditional Press Method, accessed August 7, 2025, https://researchers.westernsydney.edu.au/en/publications/fate‐of‐the‐paorlic‐compounds‐during‐olive‐oil‐production‐with‐th/fingerprints/.

[38] C. Lammi , M. Bellumori , L. Cecchi , et al., “Extra Virgin Olive Oil Phenol Extracts Exert Hypocholesterolemic Effects Through the Modulation of the LDLR Pathway: In Vitro and Cellular Mechanism of Action Elucidation,” Nutrients 12 (2020): 1723. 32526887 10.3390/nu12061723 PMC7352813

[39] A. Tomaino , M. Martorana , T. Arcoraci , D. Monteleone , C. Giovinazzo , and A. Saija , “Antioxidant Activity and Phenolic Profile of Pistachio (*Pistacia vera* L., variety Bronte) Seeds and Skins,” Biochimie 92 (2010): 1115–1122. 20388531 10.1016/j.biochi.2010.03.027

[40] M. S. Lenucci , D. Cadinu , M. Taurino , G. Piro , and G. Dalessandro , “Antioxidant Composition in Cherry and High‐Pigment Tomato Cultivars,” Journal of Agricultural and Food Chemistry 54 (2006): 2606–2613. 16569051 10.1021/jf052920c

[41] R. Tardugno , N. Cicero , R. Costa , V. Nava , and R. Vadalà , “Exploring Lignans, a Class of Health Promoting Compounds, in a Variety of Edible Oils From Brazil,” Foods 11 (2022): 1386. 35626956 10.3390/foods11101386 PMC9141677

[42] L. Siracusa , A. Saija , M. Cristani , et al., “Phytocomplexes From Liquorice (*Glycyrrhiza glabra* L.) Leaves — Chemical Characterization and Evaluation of Their Antioxidant, Anti‐Genotoxic and Anti‐Inflammatory Activity,” Fitoterapia 82 (2011): 546–556. 21281704 10.1016/j.fitote.2011.01.009

[43] S. Chelly , M. Chelly , C. Occhiuto , et al., “Evaluation of Antioxidant, Anti‐Inflammatory and Antityrosinase Potential of Extracts From Different Aerial Parts of *Rhanterium suaveolens* from Tunisia,” Chemistry and Biodiversity 18 (2021): e2100316. 34114723 10.1002/cbdv.202100316

[44] I. F. F. Benzie and J. J. Strain , “The Ferric Reducing Ability of Plasma (FRAP) as a Measure of ‘Antioxidant Power’: The FRAP Assay,” Analytical Biochemistry 239 (1996): 70–76. 8660627 10.1006/abio.1996.0292

[45] A. Dávalos , C. Gómez‐Cordovés , and B. Bartolomé , “Extending Applicability of the Oxygen Radical Absorbance Capacity (ORAC−Fluorescein) Assay,” Journal of Agricultural and Food Chemistry 52 (2004): 48–54. 14709012 10.1021/jf0305231

